# Supplementary material for: Wag31, a membrane tether, is crucial for lipid homeostasis in mycobacteria
Source: eLife. 2025 May 22;14:RP104268. doi: 10.7554/eLife.104268 (PMC12097788; doi:10.7554/eLife.104268)

Figure 1b: Source Data

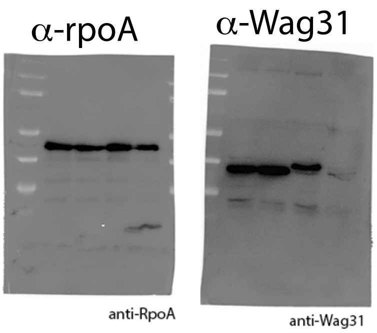

Figure 1c: Source Data

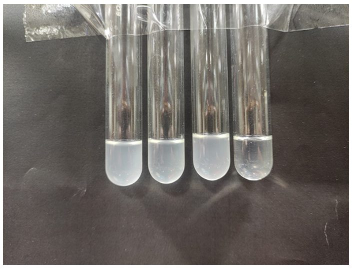

Figure 1e: Source Data

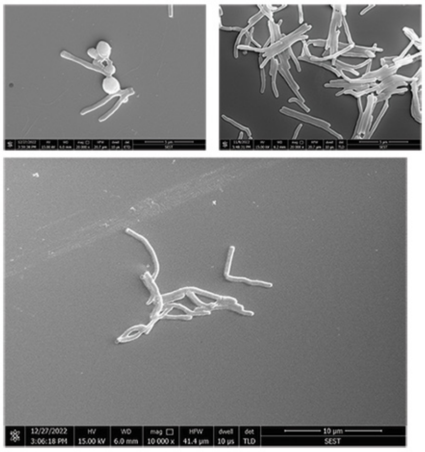

Figure 1f: Source Data

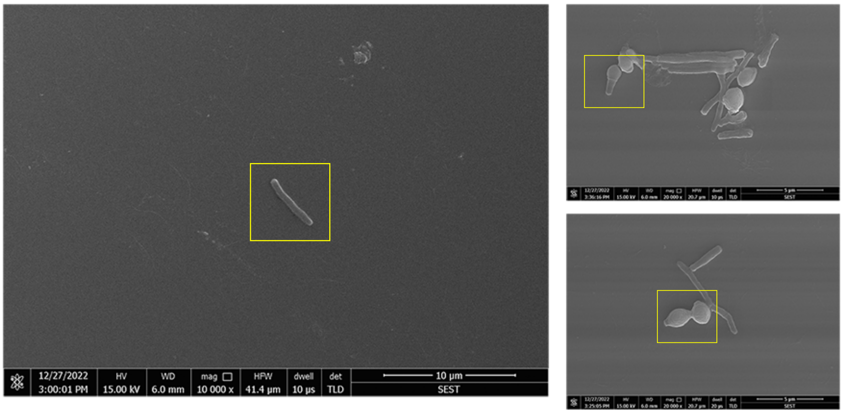

Figure 1h: Source Data

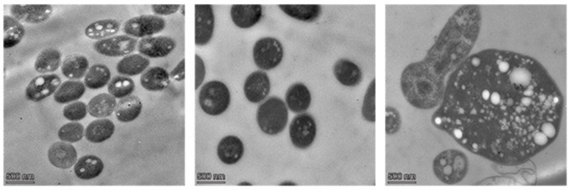

Supplement: Figure 1—source data 1. — The areas used for making the final figure are marked by yellow boxes. [file elife-104268-fig1-data1.zip › Figure 1-Source data 1.pdf]
