# Supplementary figures and images for: Wag31, a membrane tether, is crucial for lipid homeostasis in mycobacteria
Source: eLife. 2025 May 22;14:RP104268. doi: 10.7554/eLife.104268 (PMC12097788; doi:10.7554/eLife.104268)

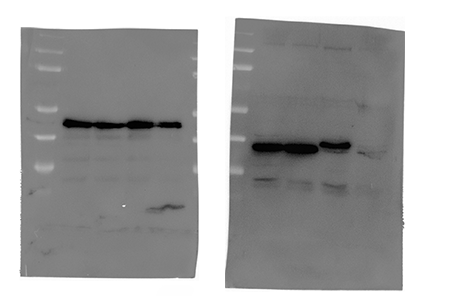

Supplement: Figure 1—source data 2. [file elife-104268-fig1-data2.zip › Figure 1-Source Data 2/Figure 1b-Source data.tif]

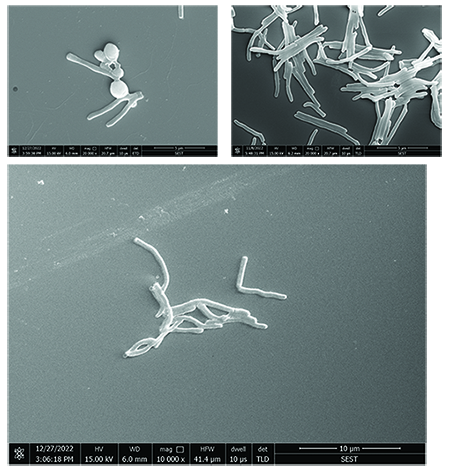

Supplement: Figure 1—source data 2. [file elife-104268-fig1-data2.zip › Figure 1-Source Data 2/Figure 1e- Source data.tif]

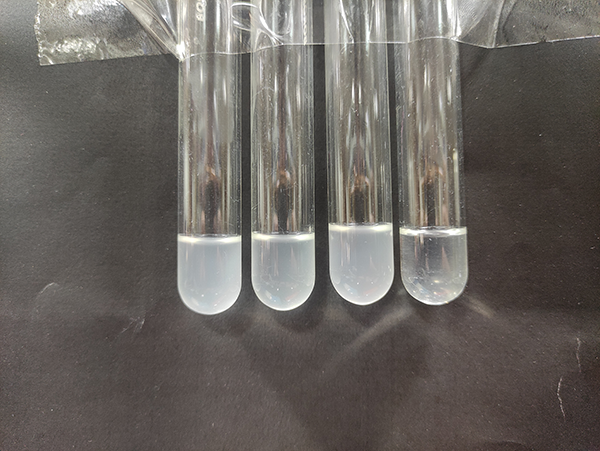

Supplement: Figure 1—source data 2. [file elife-104268-fig1-data2.zip › Figure 1-Source Data 2/Figure 1c- Source data.tif]

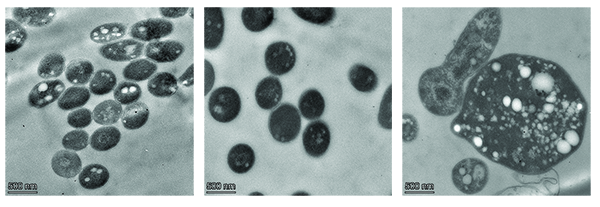

Supplement: Figure 1—source data 2. [file elife-104268-fig1-data2.zip › Figure 1-Source Data 2/Figure 1h- Source data.tif]

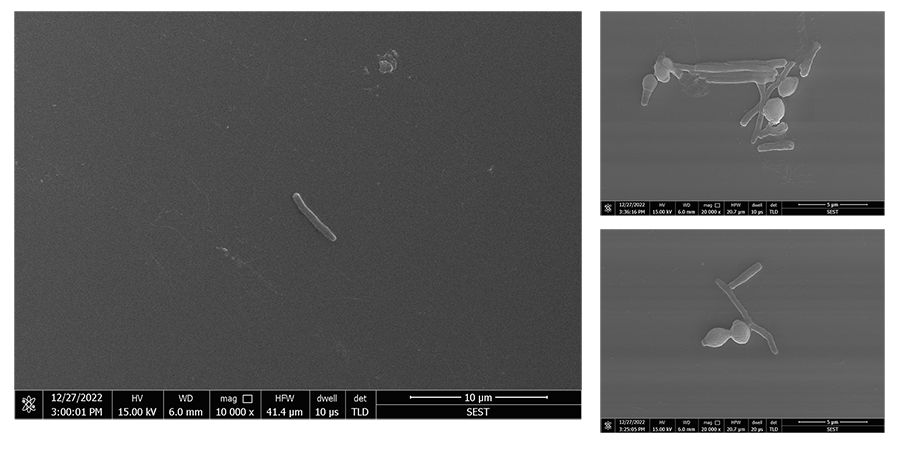

Supplement: Figure 1—source data 2. [file elife-104268-fig1-data2.zip › Figure 1-Source Data 2/Figure 1f- Source data.tif]

Source Data: Fig 1- Fig Supplement 1b: Control and other  
PCRs shown in the figure are marked.

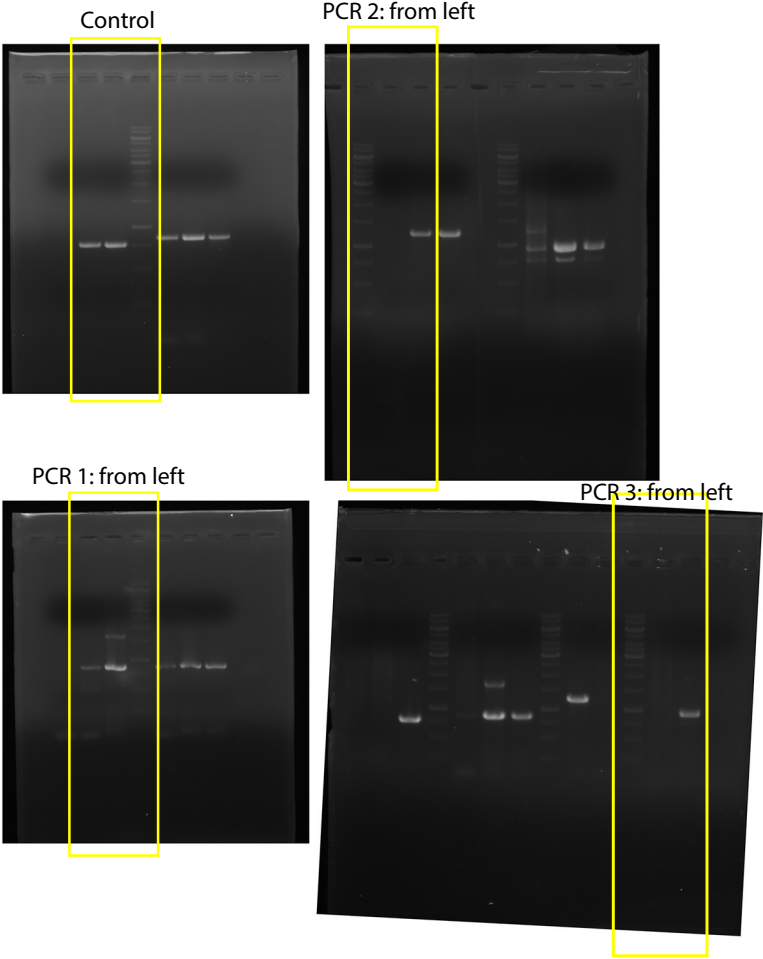

Supplement: Figure 1—figure supplement 1—source data 1. — The lanes used for making the figure are made of yellow boxes. [file elife-104268-fig1-figsupp1-data1.zip › Figure 1-Figure Supplement 1 Source Data 1.pdf]

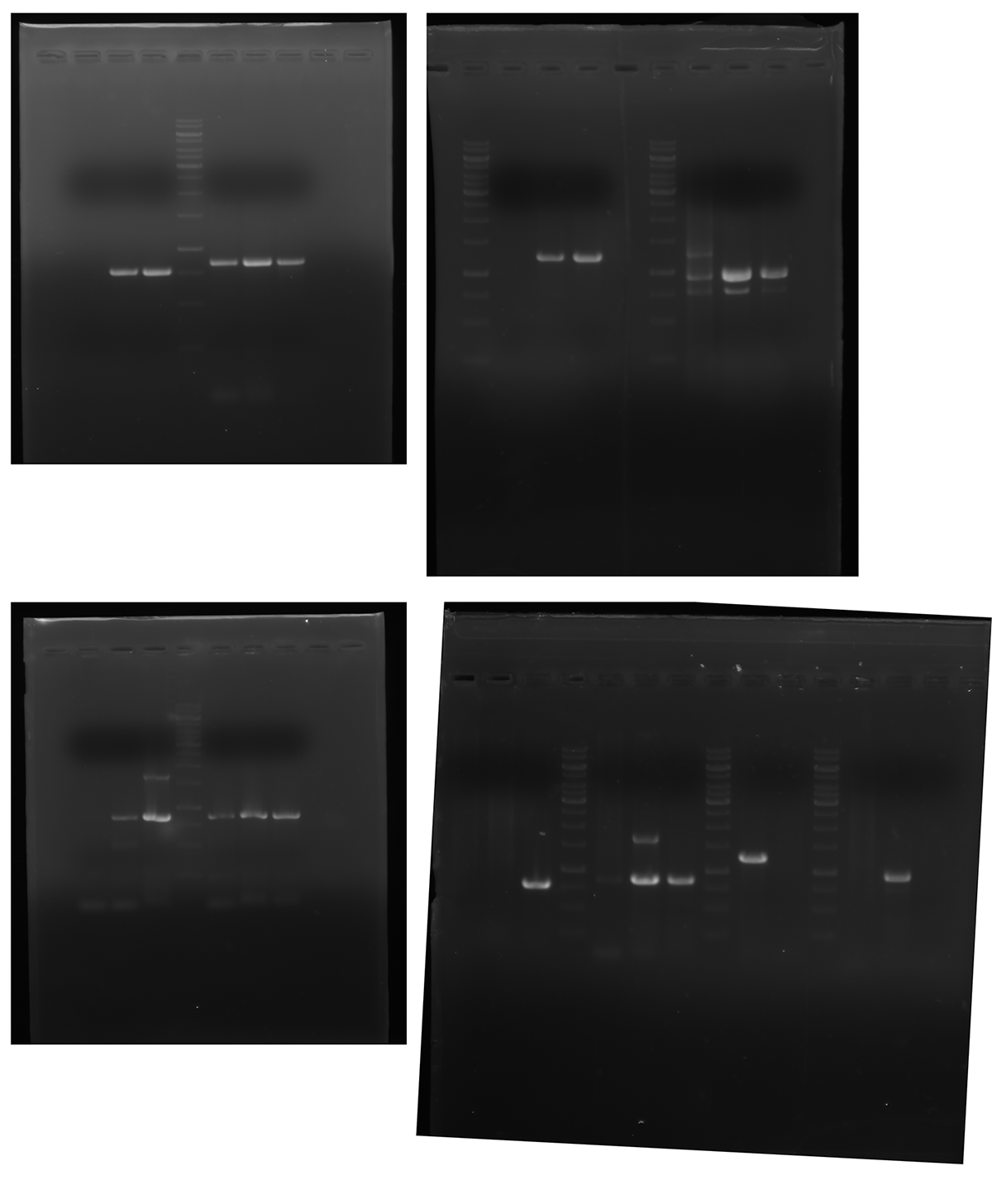

Supplement: Figure 1—figure supplement 1—source data 2. [file elife-104268-fig1-figsupp1-data2.zip › Figure 1-Figure Supplement 1-Source Data 2/Figure 1- Figure Supplement 1.tif]

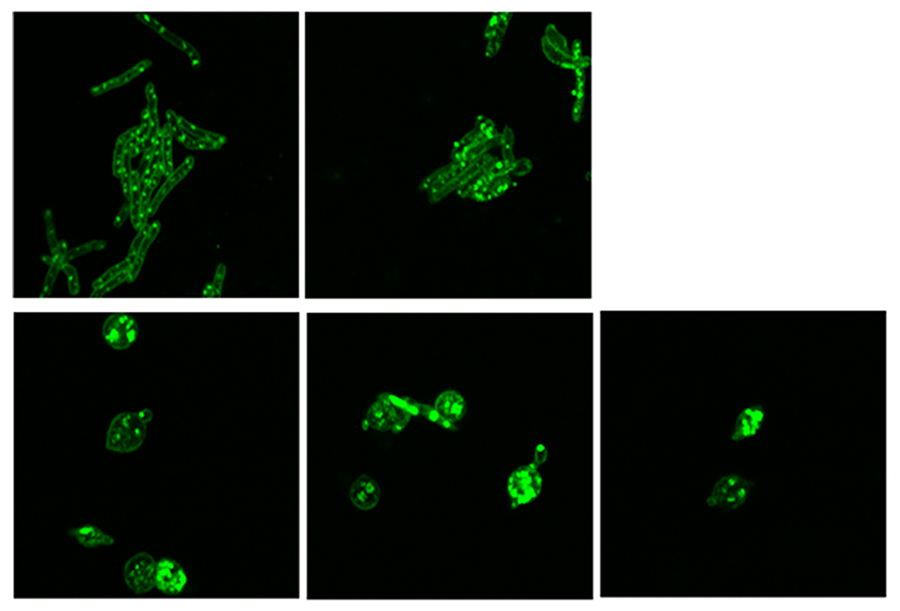

Supplement: Figure 2—source data 2. [file elife-104268-fig2-data2.zip › Figure 2-Source Data 2/Figure 2a- Source data.tif]

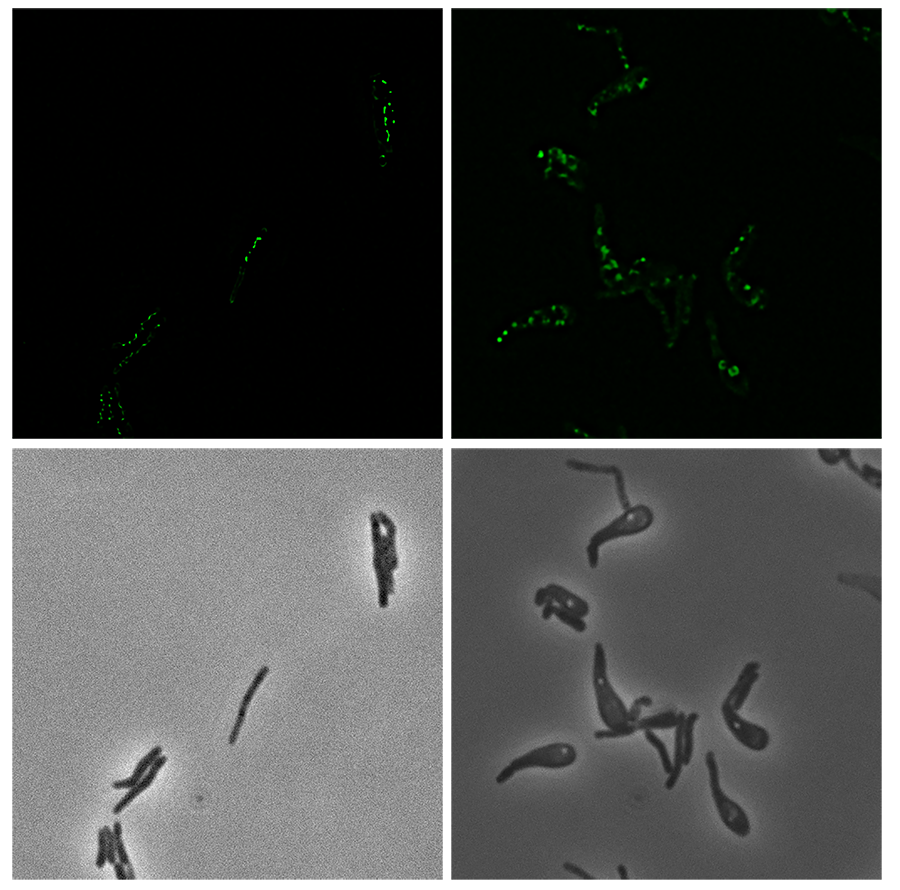

Supplement: Figure 2—source data 2. [file elife-104268-fig2-data2.zip › Figure 2-Source Data 2/Figure 2b- Source data.tif]

Figure 2-Figure Supplement 1-Source Data 1

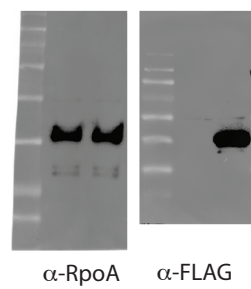

Supplement: Figure 2—figure supplement 1—source data 1. [file elife-104268-fig2-figsupp1-data1.zip › Figure 2-Figure Supplement 1-Source Data 1.pdf]

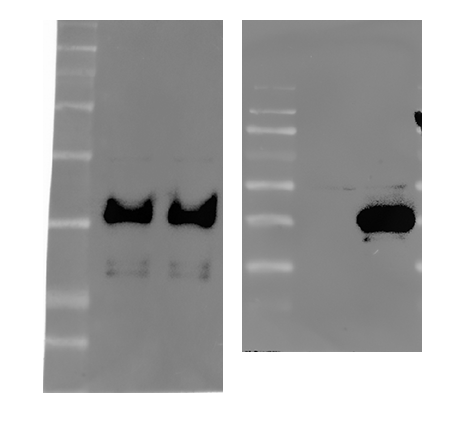

Supplement: Figure 2—figure supplement 1—source data 2. [file elife-104268-fig2-figsupp1-data2.zip › Figure 2-Figure Supplement 1-Source Data 2/Figure2-Figure supplement 1-Source data2.tif]

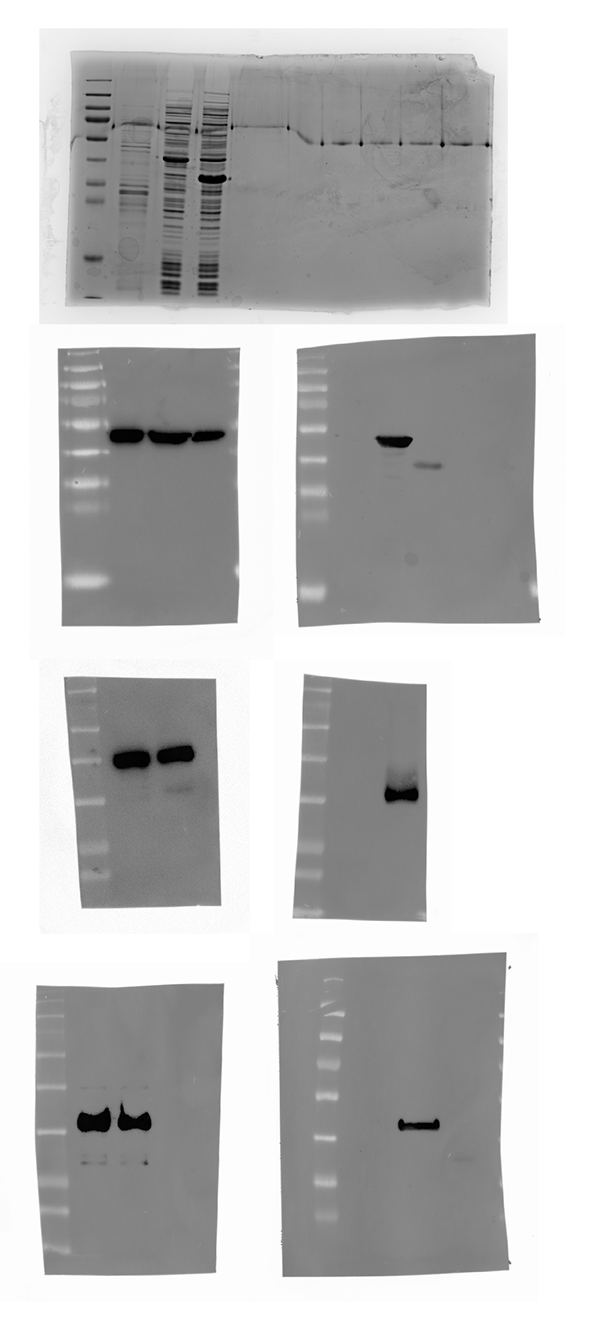

Supplement: Figure 3—source data 2. [file elife-104268-fig3-data2.zip › Figure 3-Source Data 3/Figure 3b,c.tif]

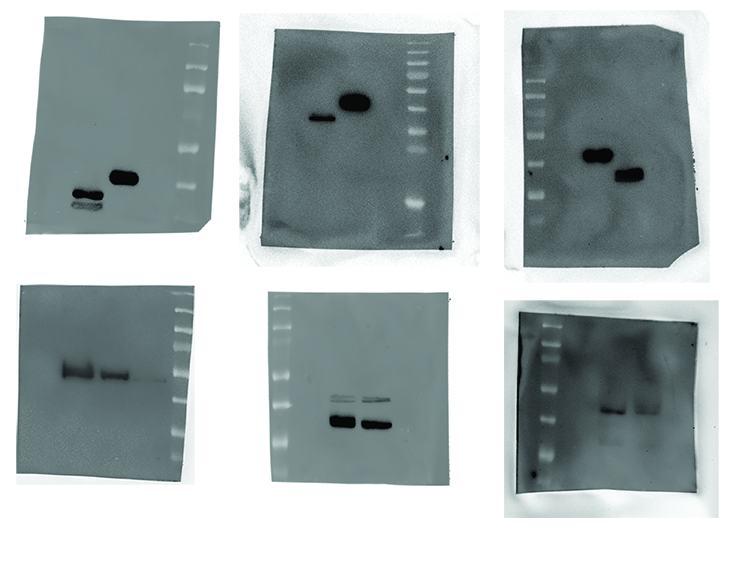

Supplement: Figure 3—source data 2. [file elife-104268-fig3-data2.zip › Figure 3-Source Data 3/Figure 3d- Source data.tif]

Figure 3- Source Data 2- Source data used for generating 3b, 3c and 3d

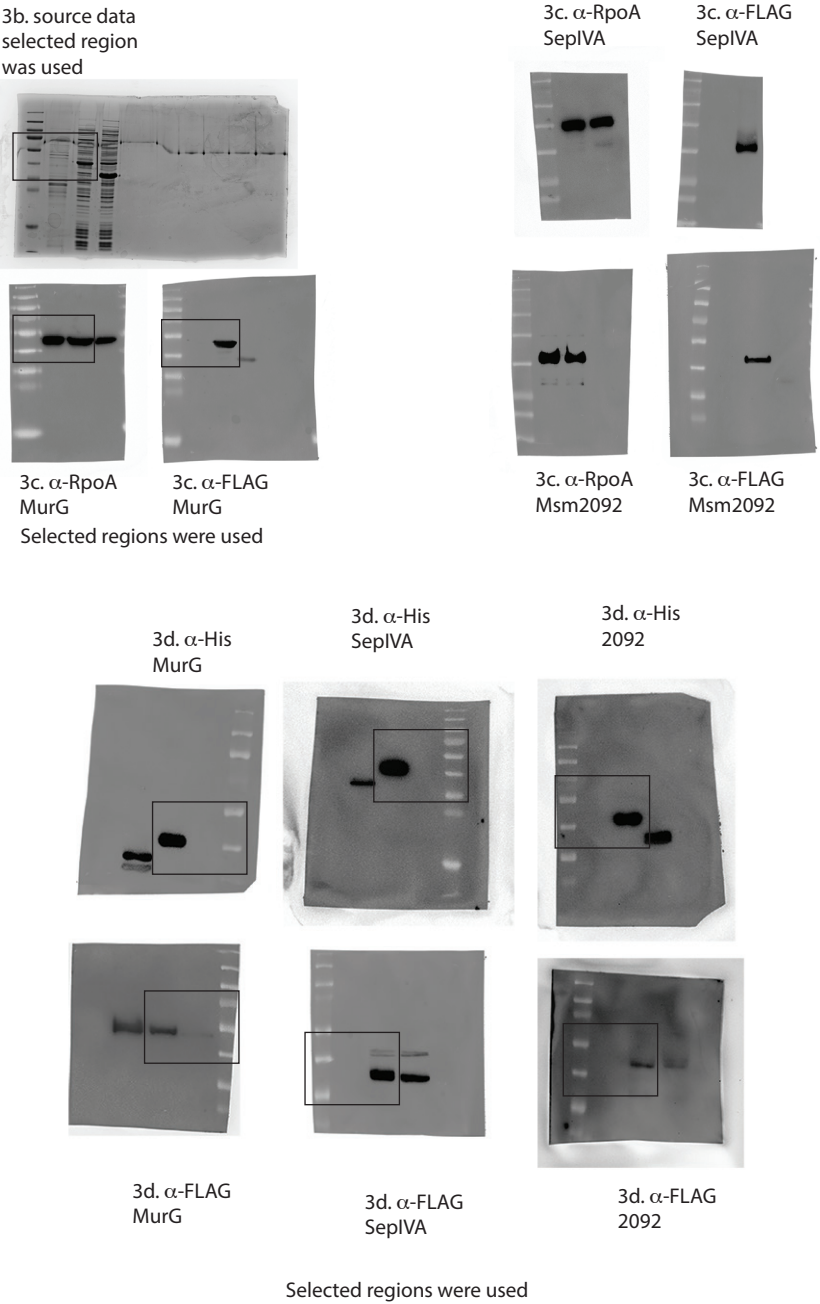

Supplement: Figure 3—source data 3. — Areas used for making the figure are marked. [file elife-104268-fig3-data3.zip › Figure 3-Source Data 2.pdf]

Figure 3-Figure Supplement 1- Source Data

Figure 3-Figure Supplement 1a- Source Data

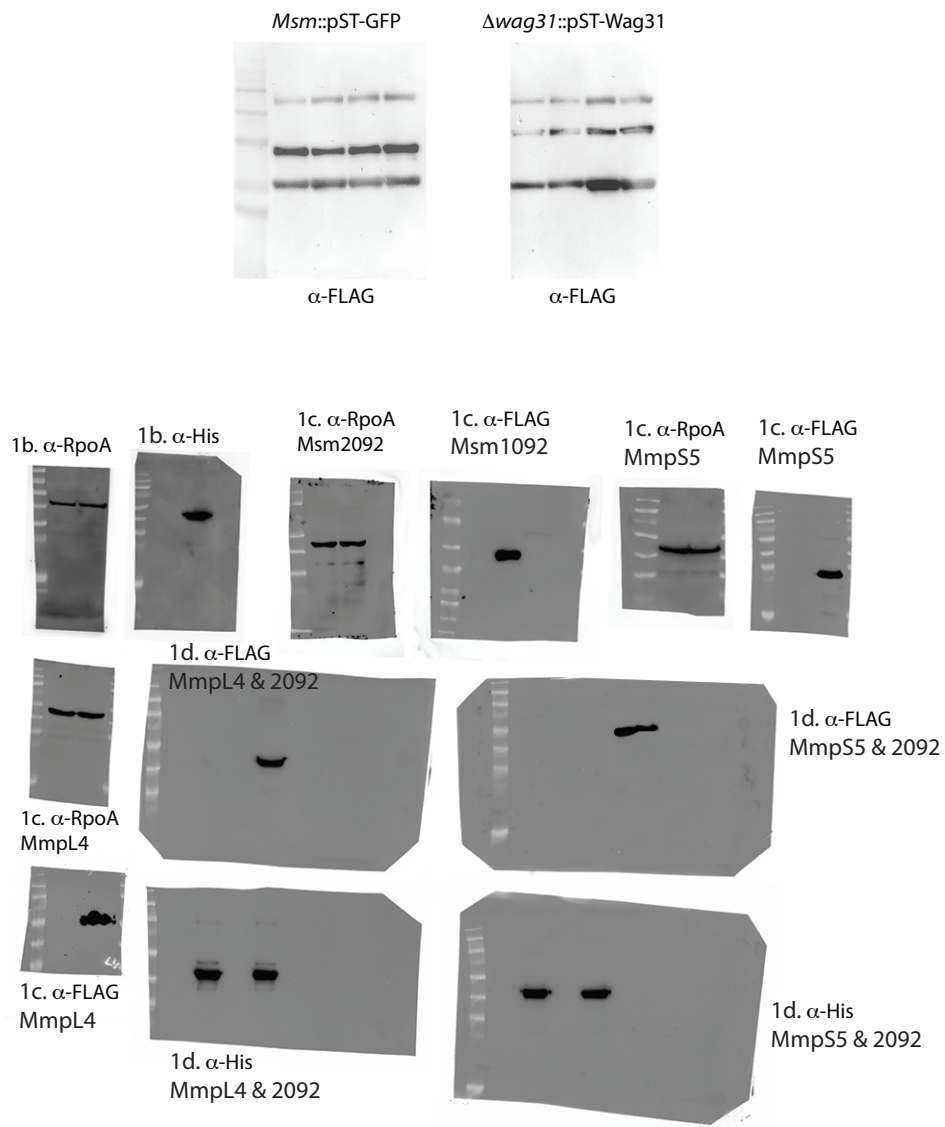

Supplement: Figure 3—figure supplement 1—source data 1. [file elife-104268-fig3-figsupp1-data1.zip › Figure 3-Figure Supplement 1-Source Data 1.pdf]

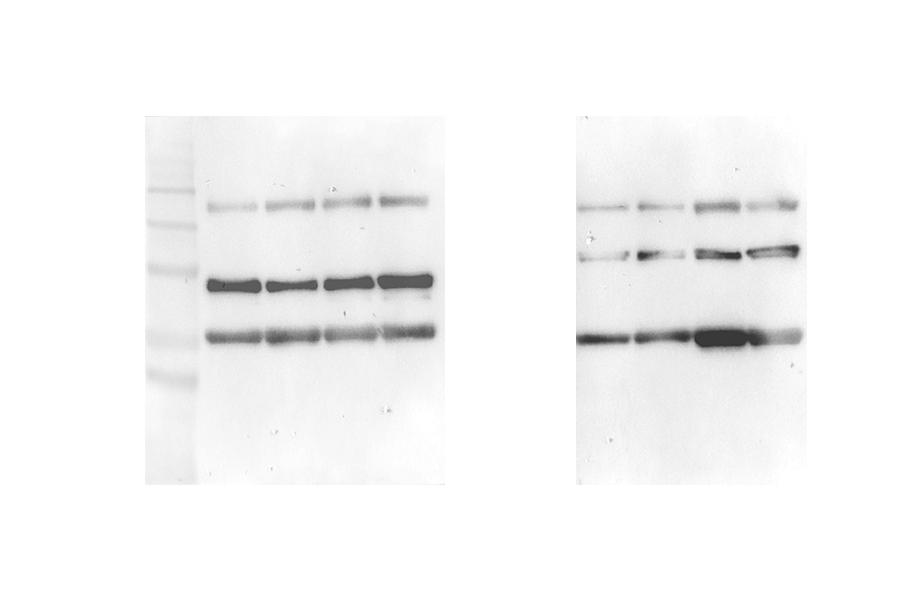

Supplement: Figure 3—figure supplement 1—source data 2. [file elife-104268-fig3-figsupp1-data2.zip › Figure 3-Figure Supplement 1-Source Data 2/Figure 3-Figure supplement 1a-Source data.tif]

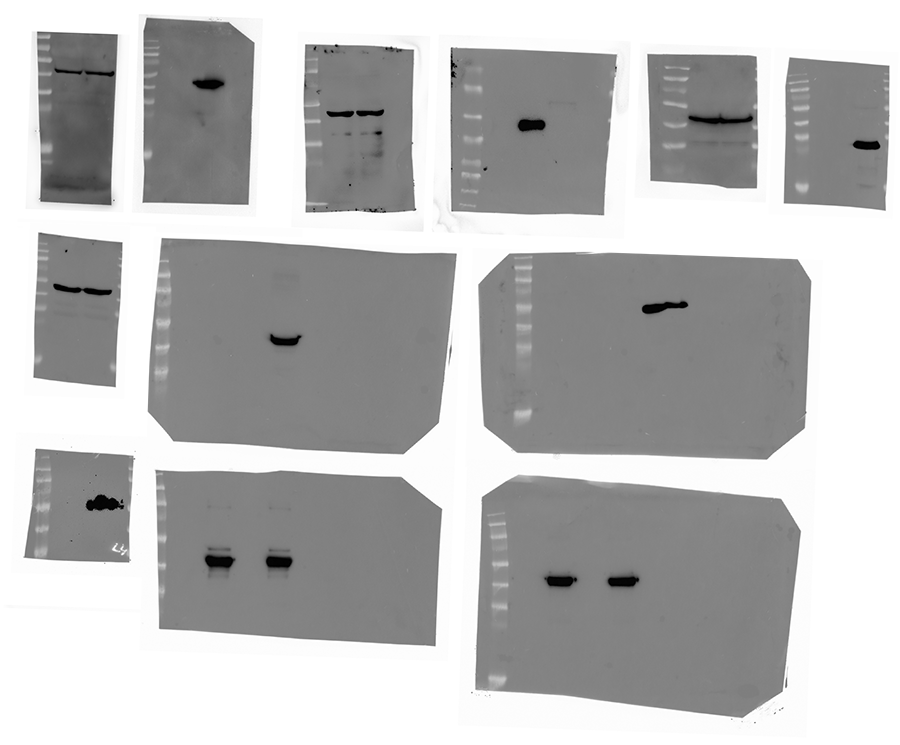

Supplement: Figure 3—figure supplement 1—source data 2. [file elife-104268-fig3-figsupp1-data2.zip › Figure 3-Figure Supplement 1-Source Data 2/Figure3- Figure supplement 1-b-d- Source data.tif]

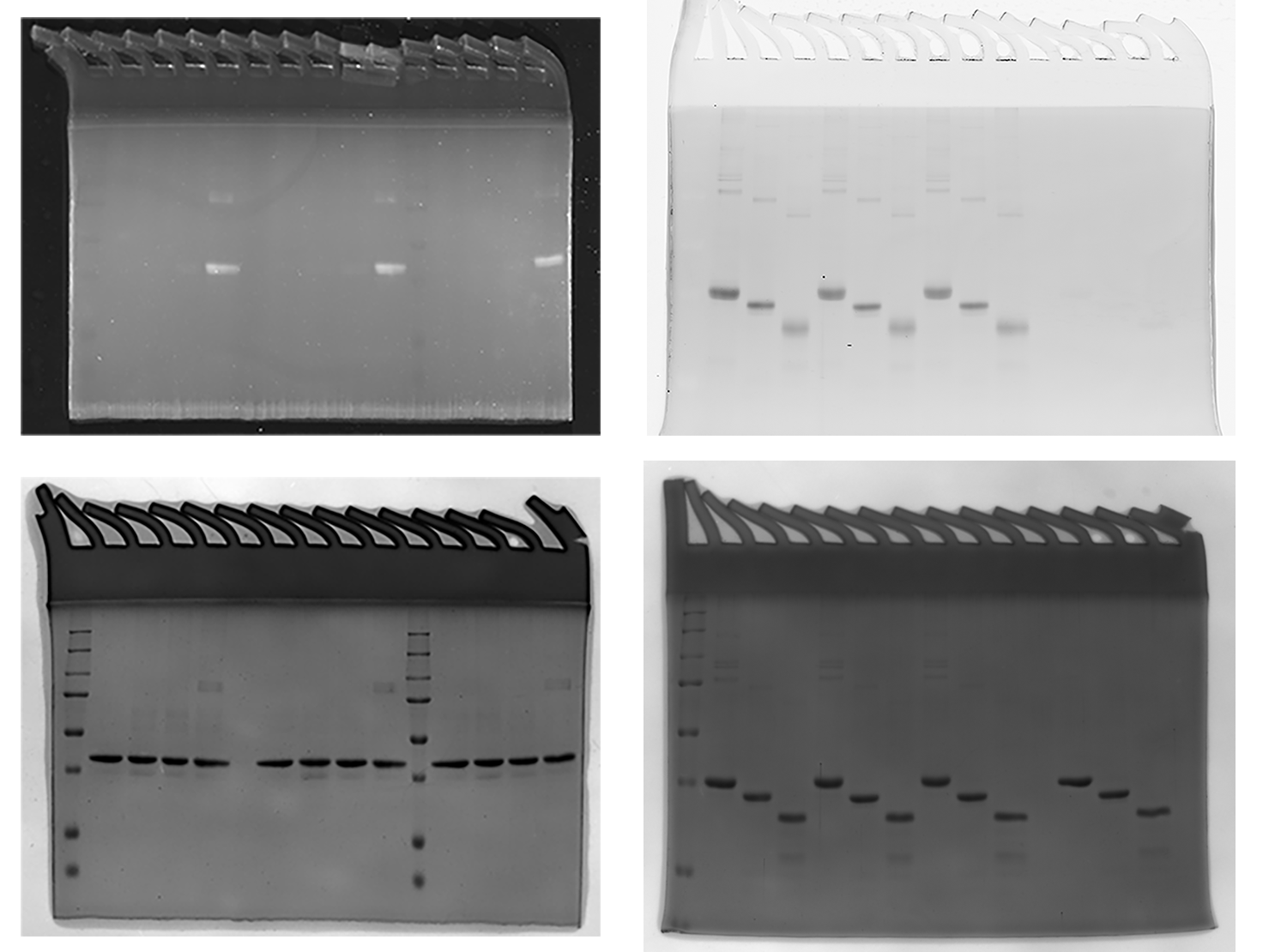

Supplement: Figure 4—source data 2. [file elife-104268-fig4-data2.zip › Figure 4-Source Data 2/Figure 4b,c-Source Data 1.tif]

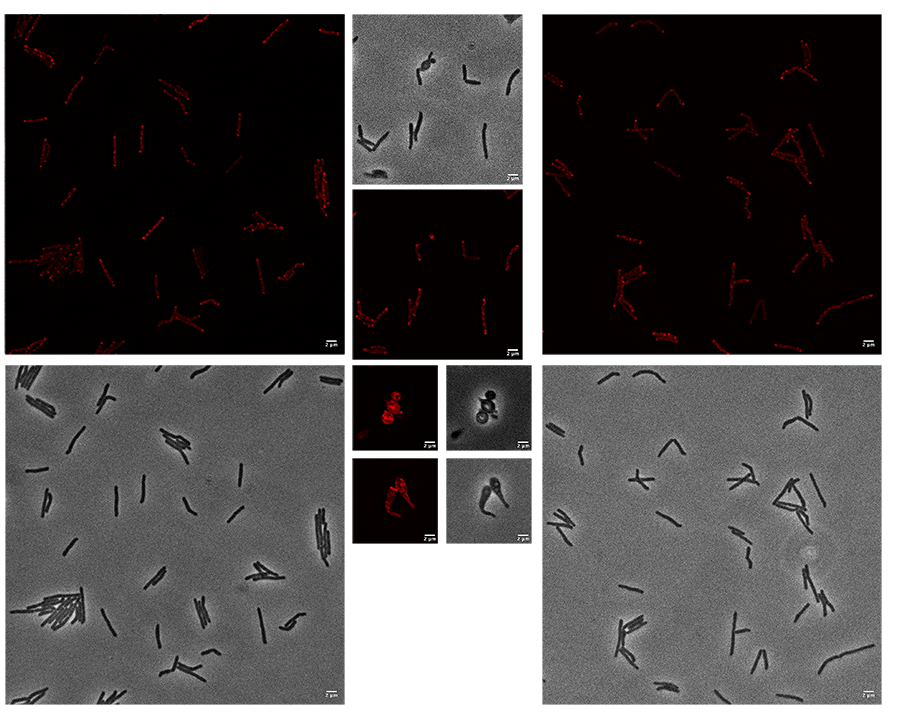

Supplement: Figure 5—source data 2. [file elife-104268-fig5-data2.zip › Figure 5-Source Data 2/Figure 5-Source data 1.tif]

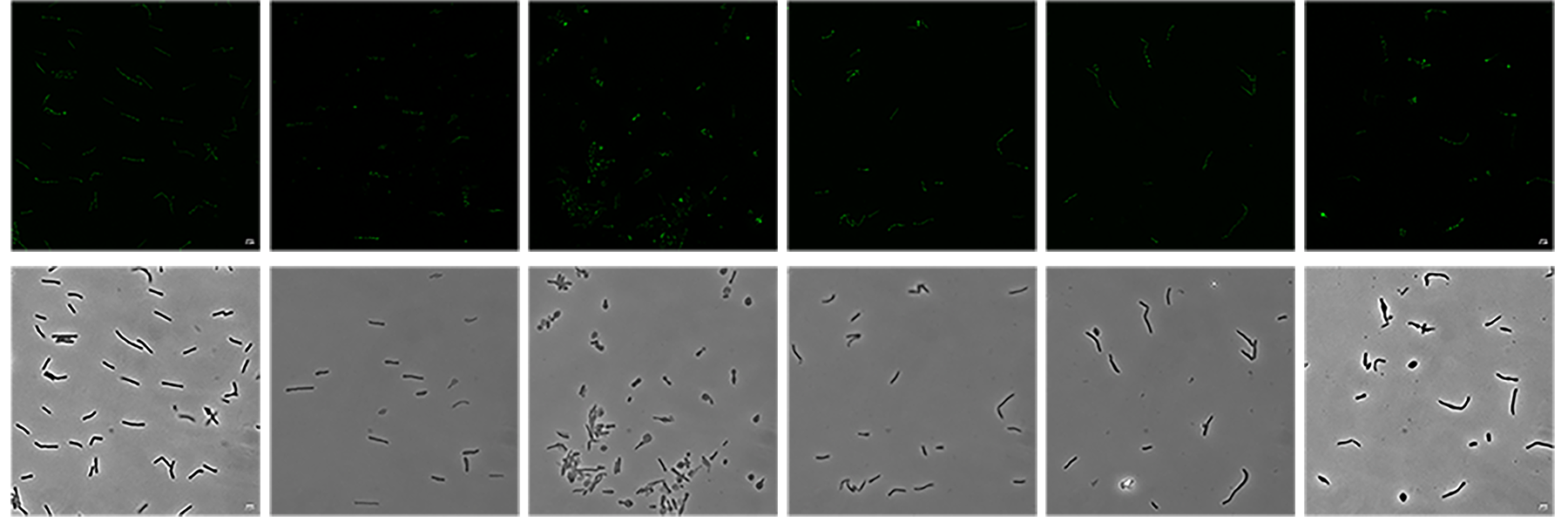

Supplement: Figure 6—source data 2. [file elife-104268-fig6-data2.zip › Figure 6-Source Data 2/Figure 6a- Source data.tif]

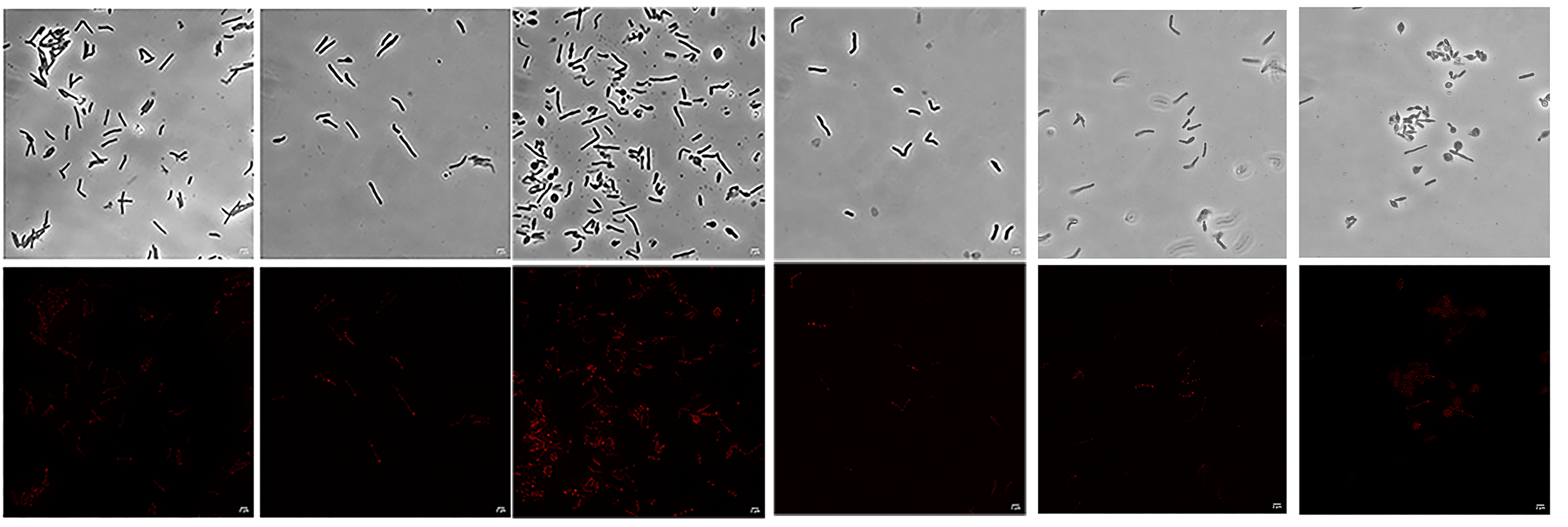

Supplement: Figure 6—source data 2. [file elife-104268-fig6-data2.zip › Figure 6-Source Data 2/Figure 6c- Source data.tif]

Figure 7-Source Data 1

Figure 7b. Selected region was used for the figure

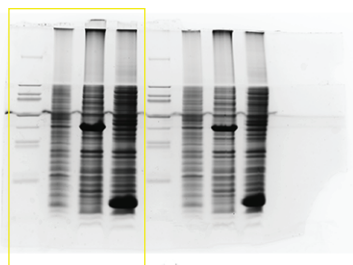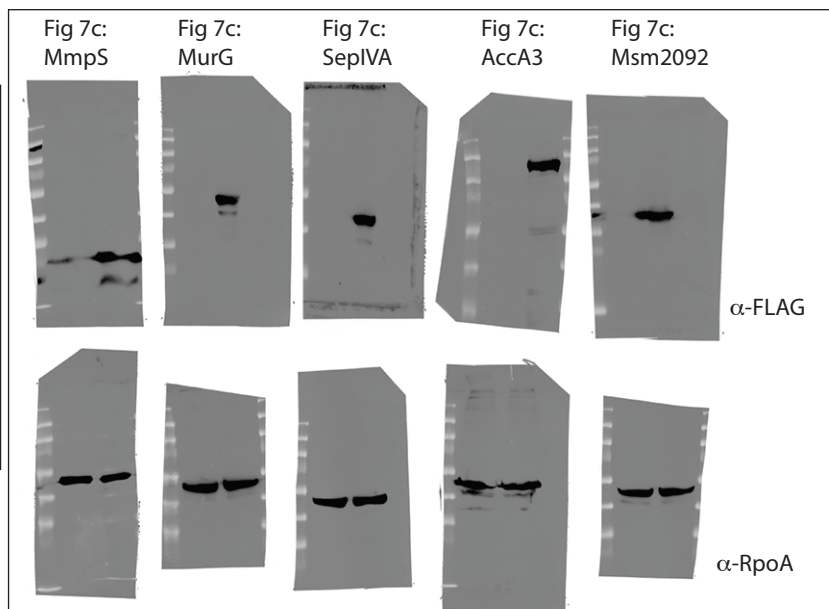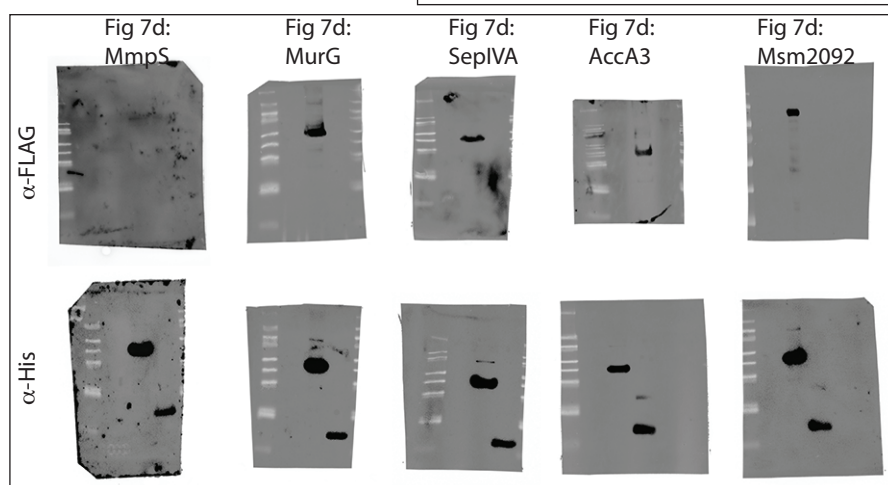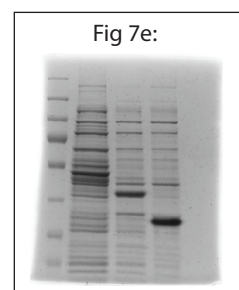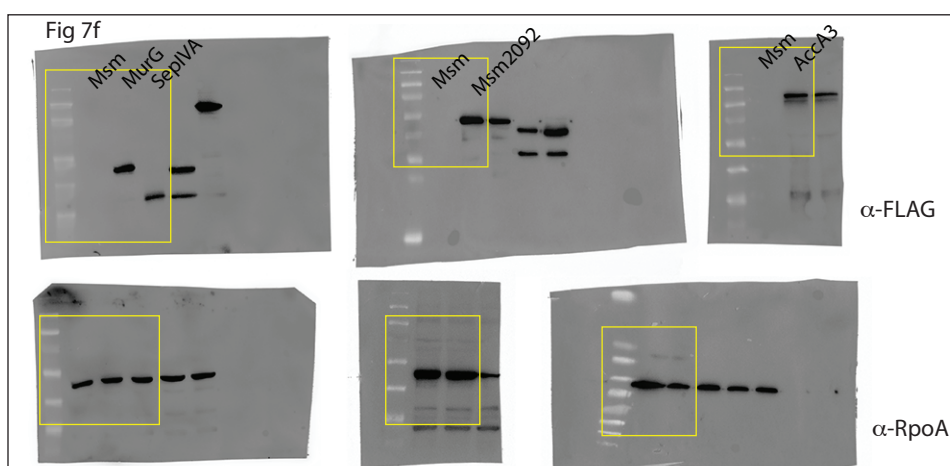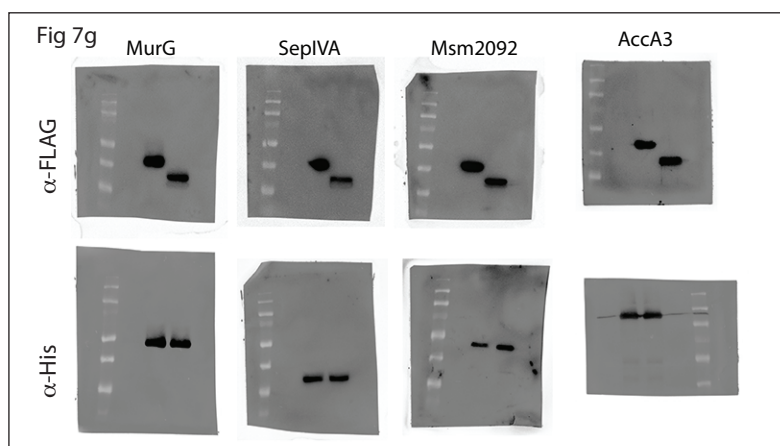

Supplement: Figure 7—source data 1. — The lanes used to make the panels are marked. [file elife-104268-fig7-data1.zip › Figure 7-Source Data 1.pdf]

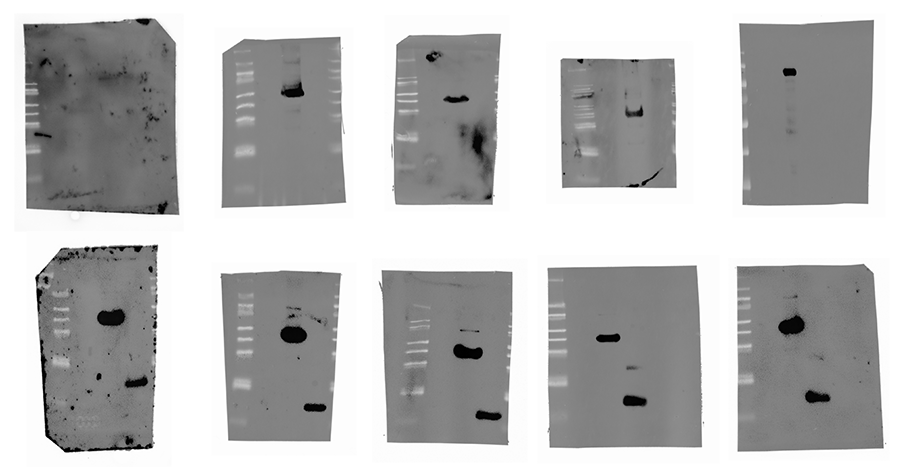

Supplement: Figure 7—source data 2. [file elife-104268-fig7-data2.zip › Figure 7-Source Data 2/Figure 7d- Source data_nolablel.tif]

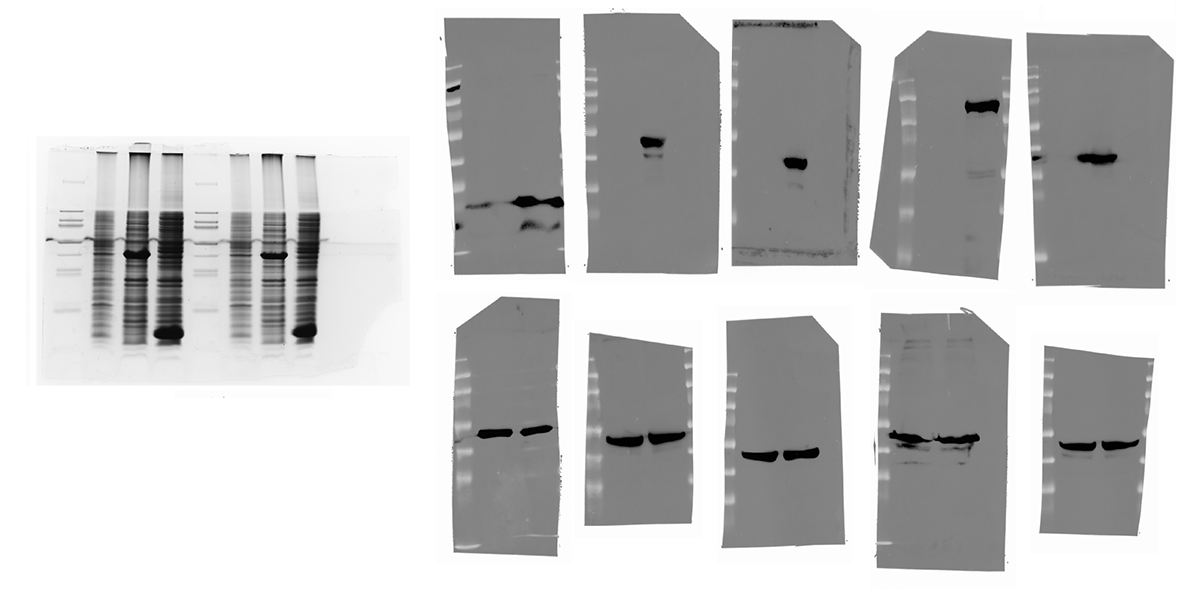

Supplement: Figure 7—source data 2. [file elife-104268-fig7-data2.zip › Figure 7-Source Data 2/Figure 7b,c- Source data_nolabel.tif]

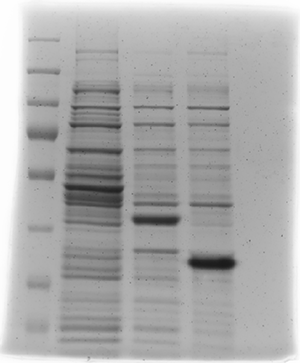

Supplement: Figure 7—source data 2. [file elife-104268-fig7-data2.zip › Figure 7-Source Data 2/Figure 7e- Source data.tif]

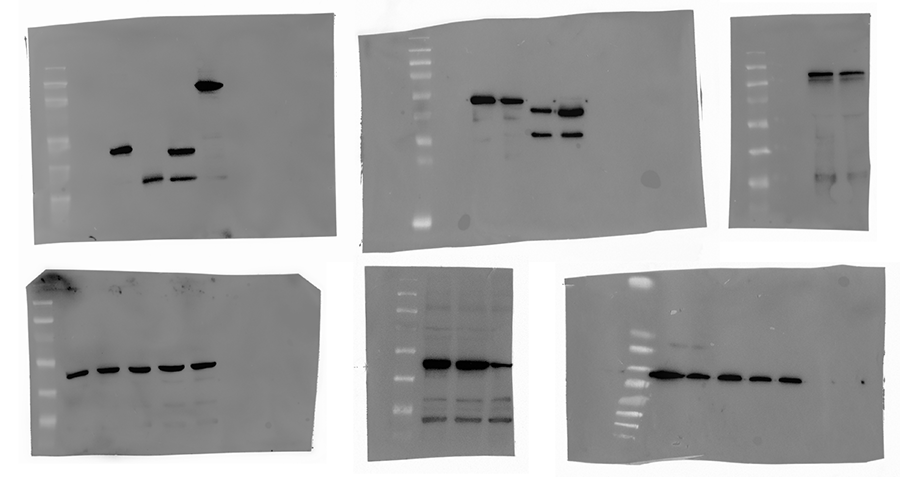

Supplement: Figure 7—source data 2. [file elife-104268-fig7-data2.zip › Figure 7-Source Data 2/Figure 7f- Source data_nolabel.tif]

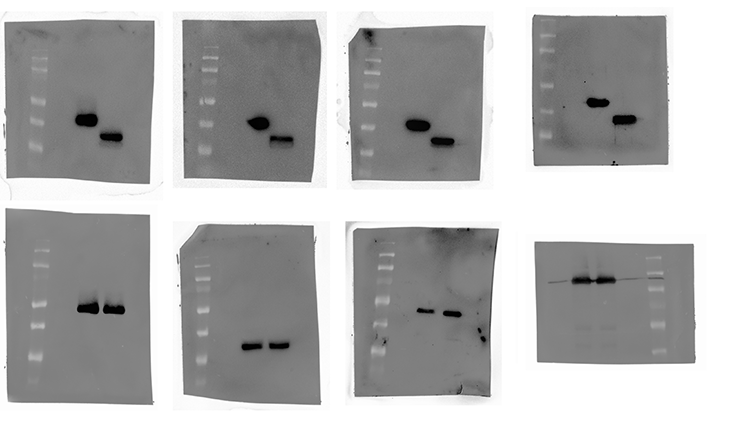

Supplement: Figure 7—source data 2. [file elife-104268-fig7-data2.zip › Figure 7-Source Data 2/Figure 7g-Source data_nolabel.tif]
