# Supplementary material for: Wag31, a membrane tether, is crucial for lipid homeostasis in mycobacteria
Source: eLife. 2025 May 22;14:RP104268. doi: 10.7554/eLife.104268 (PMC12097788; doi:10.7554/eLife.104268)

Figure 2a-Source Data- Areas used for making the final figure are marked

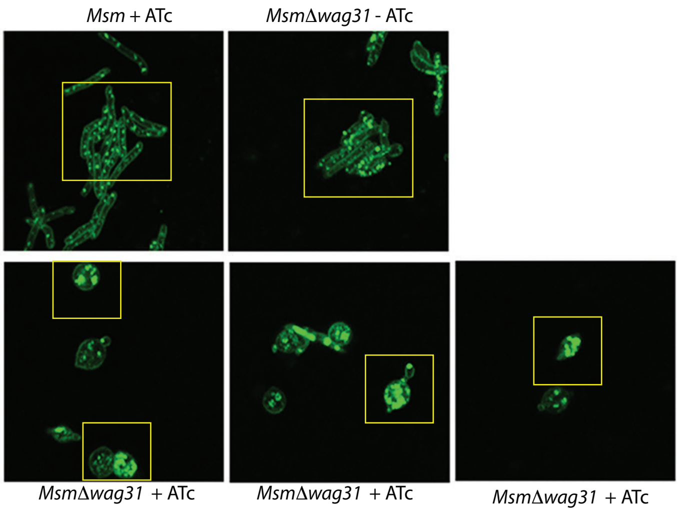

Figure 2b-Source Data: areas used for making the final figure are marked

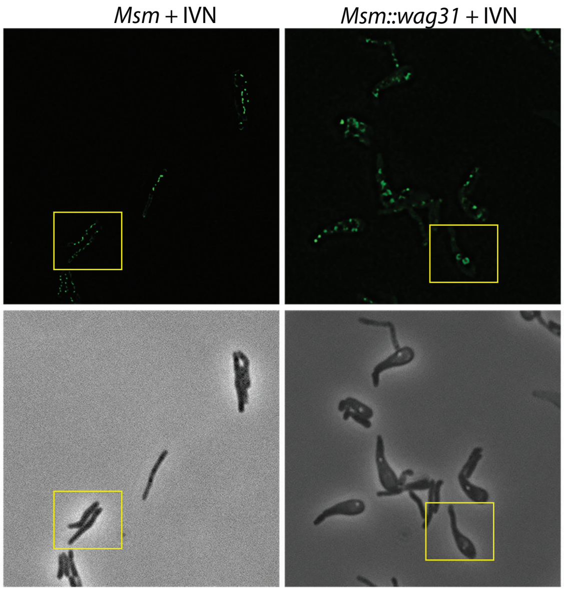

Supplement: Figure 2—source data 1. — The areas used for making the figure are marked. [file elife-104268-fig2-data1.zip › Figure 2-Source Data 1.pdf]
