# Supplementary material for: Wag31, a membrane tether, is crucial for lipid homeostasis in mycobacteria
Source: eLife. 2025 May 22;14:RP104268. doi: 10.7554/eLife.104268 (PMC12097788; doi:10.7554/eLife.104268)
Supplement: Figure 4—source data 1. — Areas used for making the figure are marked. [file elife-104268-fig4-data1.zip › Figure 4-Source Data 1.pdf]

Figure 4-Source Data 1

Figure 4b: Source Data

Fluorescence

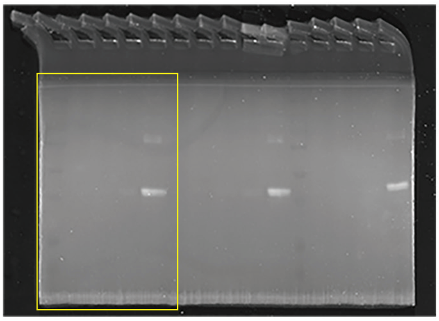

Figure 4c: Source Data

Fluorescence

CL-liposomes no liposomes

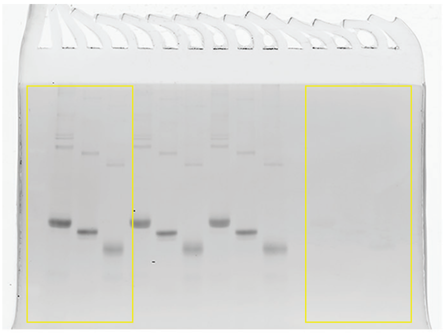

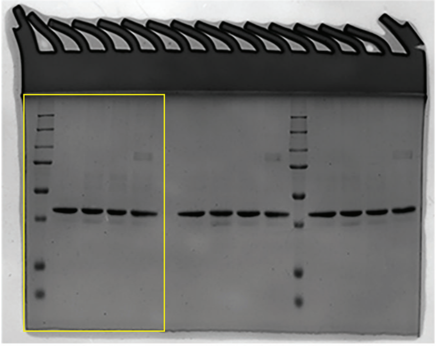

Coomassie

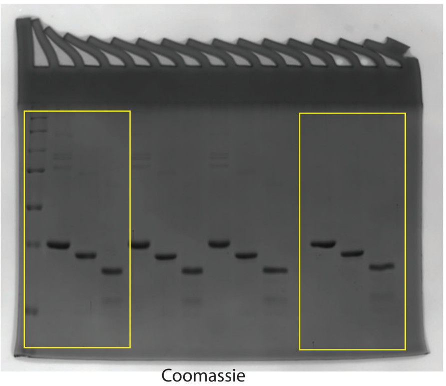

Coomassie

Regions marked were used for making the Figure
