# Supplementary material for: Wag31, a membrane tether, is crucial for lipid homeostasis in mycobacteria
Source: eLife. 2025 May 22;14:RP104268. doi: 10.7554/eLife.104268 (PMC12097788; doi:10.7554/eLife.104268)
Supplement: Figure 5—source data 1. — Areas used for making the figure are marked. [file elife-104268-fig5-data1.zip › Figure 5-Source data 1.pdf]

## Figure 5a-Source Data

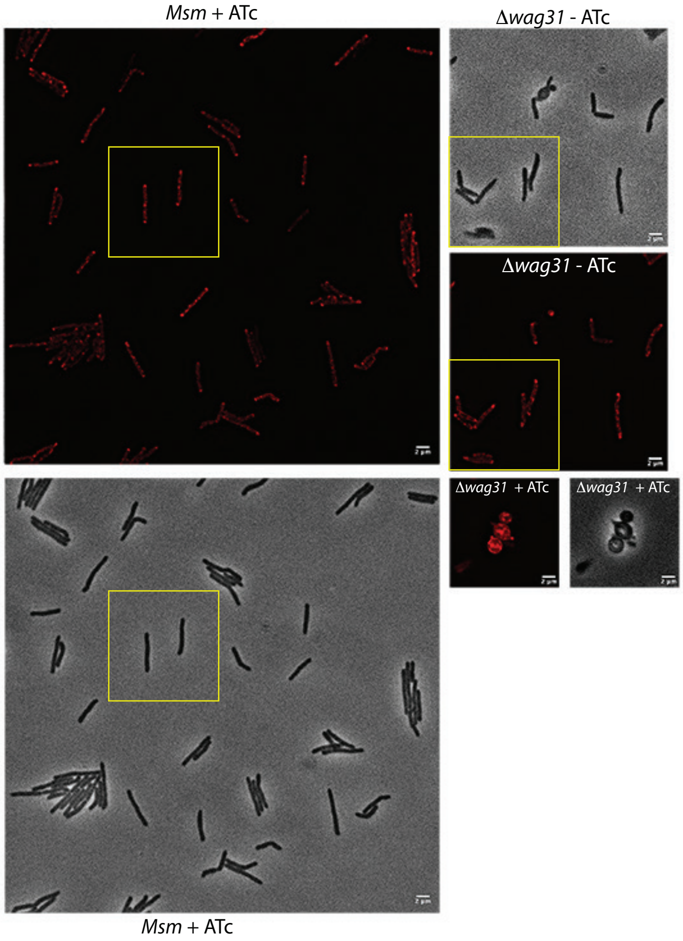


Boxed areas were used for the figure

## Figure 5b-Source Data

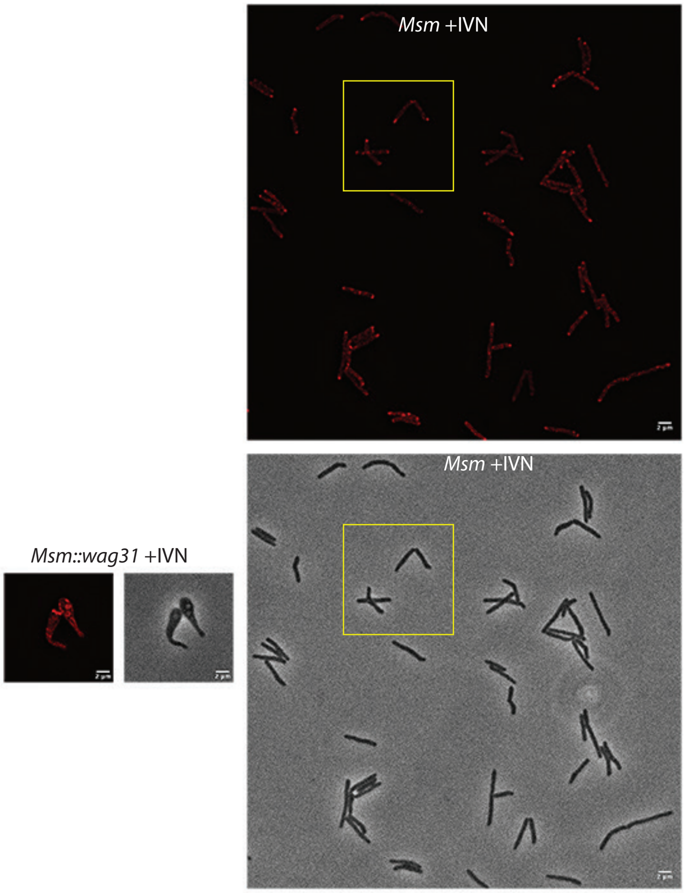


Boxed areas were used for the figure
