# Supplementary material for: Wag31, a membrane tether, is crucial for lipid homeostasis in mycobacteria
Source: eLife. 2025 May 22;14:RP104268. doi: 10.7554/eLife.104268 (PMC12097788; doi:10.7554/eLife.104268)

Figure 6a-Source Data- Area shown by yellow box is used for making the figure

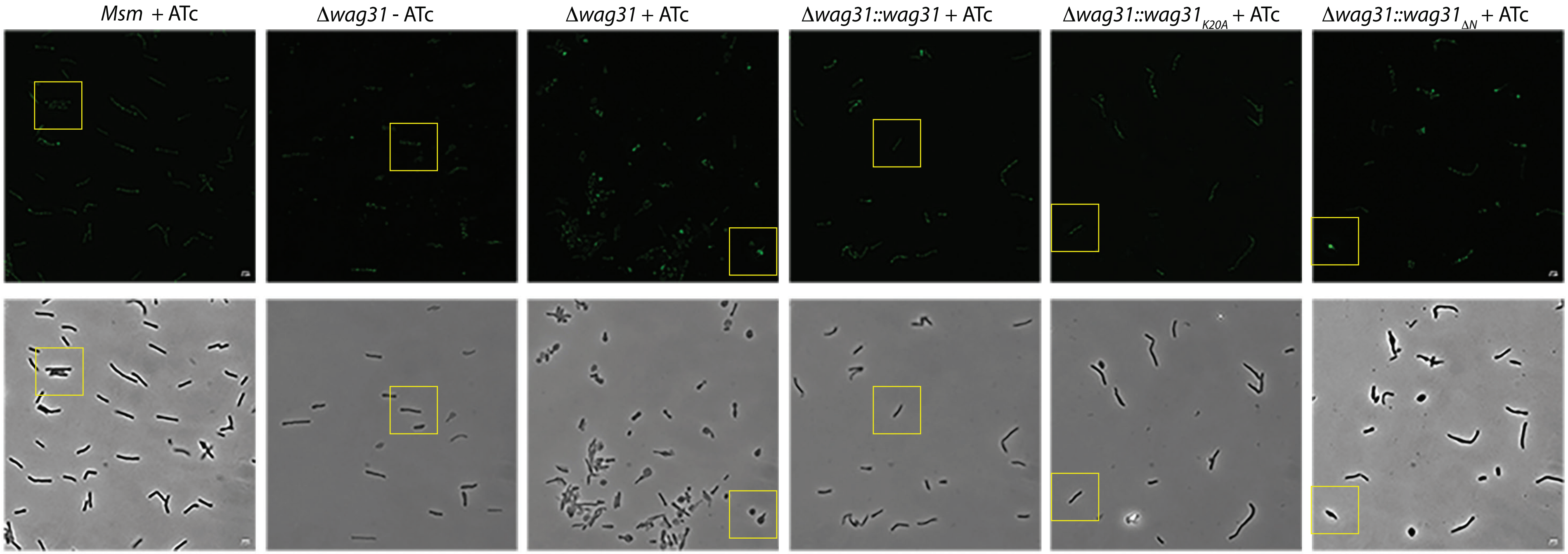

Figure 6c-Source Data- Area shown by yellow box is used for making the figure

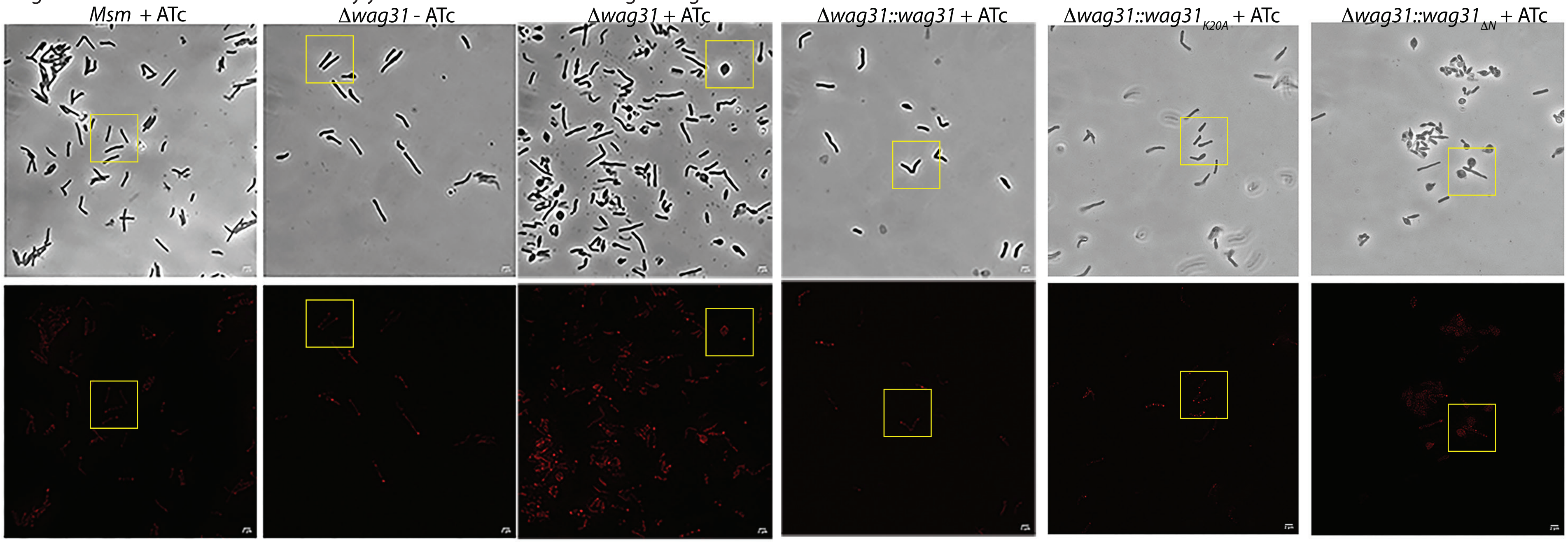

Supplement: Figure 6—source data 1. — The areas used for making the figure panels are marked. [file elife-104268-fig6-data1.zip › Figure 6-Source Data 1.pdf]
